# Adhesins as targets for vaccine development.

**DOI:** 10.3201/eid0503.990310

**Published:** 1999

**Authors:** T M Wizemann, J E Adamou, S Langermann

**Affiliations:** MedImmune, Inc., Gaithersburg, Maryland 20878, USA.

## Abstract

Blocking the primary stages of infection, namely bacterial attachment to host cell receptors and colonization of the mucosal surface, may be the most effective strategy to prevent bacterial infections. Bacterial attachment usually involves an interaction between a bacterial surface protein called an adhesin and the host cell receptor. Recent preclinical vaccine studies with the FimH adhesin (derived from uropathogenic Escherichia coli) have confirmed that antibodies elicited against an adhesin can impede colonization, block infection, and prevent disease. The studies indicate that prophylactic vaccination with adhesins can block bacterial infections. With recent advances in the identification, characterization, and isolation of other adhesins, similar approaches are being explored to prevent infections, from otitis media and dental caries to pneumonia and sepsis.


### Adhesins as Targets for Vaccine Development

**Theresa M. Wizemann, John E. Adamou, and Solomon Langermann**
MedImmune, Inc., Gaithersburg, Maryland, USA

Blocking the primary stages of infection, namely bacterial attachment to host cell receptors and colonization of the mucosal surface, may be the most effective strategy to prevent bacterial infections. Bacterial attachment usually involves an interaction between a bacterial surface protein called an adhesin and the host cell receptor. Recent preclinical vaccine studies with the FimH adhesin (derived from uropathogenic *Escherichia coli*) have confirmed that antibodies elicited against an adhesin can impede colonization, block infection, and prevent disease. The studies indicate that prophylactic vaccination with adhesins can block bacterial infections. With recent advances in the identification, characterization, and isolation of other adhesins, similar approaches are being explored to prevent infections, from otitis media and dental caries to pneumonia and sepsis.

The ultimate aim of any vaccine is to produce long-term protective immune responses against a pathogen. These responses include systemic humoral antibodies that neutralize invasive organisms and cytotoxic T cells, which are required to clear certain infections, particularly chronic viral infections such as HIV (1). Helper T-cell and cytokine responses also influence humoral and cellular immune responses (2). For most bacteria and viruses, the first encounter with their host involves attachment to a eukaryotic cell surface, which results in colonization of the host prior to disease. In such cases, induced antibody responses at the mucosal surface could prevent attachment and abrogate colonization. The ideal target for such antibodies—surface proteins known as adhesins—mediate microbial attachment to host tissue (3). We review recent advances in the identification, isolation, and purification of adhesins (and putative adhesins) that could serve as vaccines to elicit such responses. Studies of at least one adhesin, the FimH protein from uropathogenic *Escherichia coli*, show that anti–adhesin antibodies can block microbial attachment and subsequent disease. Furthermore, while specific induction of immune responses along the mucosal surface by mucosal immunization may have its advantages (4-7), the FimH studies demonstrate that immunoglobulin (Ig) G antibodies alone, which transudate into secretions after parenteral vaccination with the FimH adhesin, are sufficient to block colonization and infection.

Address for correspondence: Solomon Langermann, MedImmune, Inc., Department of Immunology and Molecular Genetics, 35 West Watkins Mill Road, Gaithersburg, MD 20878, USA; fax: 301-527-4200; e-mail: langermanns@medimmune.com.

### The Role of Adhesins in Microbial Pathogenesis

To initiate infection, bacterial pathogens must first be able to colonize an appropriate target tissue of the host (8,9). This tropism (ability to gain access to a niche within the body), in association with the ability of the bacterium to breach mucosal barriers and invade the host, distinguishes pathogenic from commensal organisms. Colonization begins with the attachment of the bacterium to receptors expressed by cells forming the lining of the mucosa (Figure 1A, 1B). Certain species of bacteria are restricted in terms of the hosts and tissues they infect and the diseases they cause. In many cases, tropism for specific tissues has been corroborated in the laboratory by in vitro binding assays with isolated epithelial cells collected from sites of infection or from infection-prone hosts.

Attachment is mediated by adhesin proteins; bacterial lectins are the most common type of adhesin among both gram-negative and gram-positive bacteria (3,10-12). Adhesins, such as the

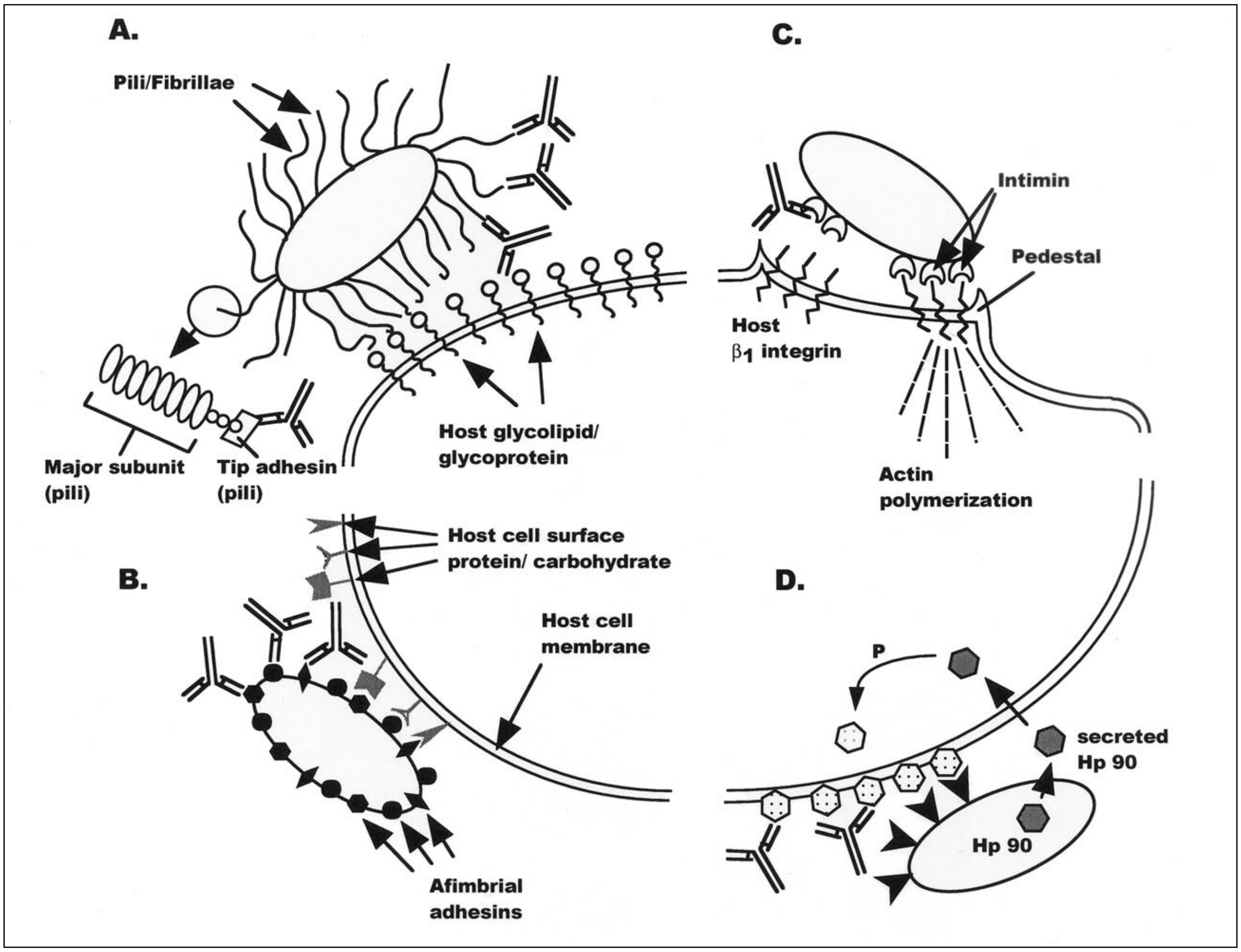

Figure 1. Four mechanisms of bacterial adherence where anti-adhesin vaccines could potentially block colonization and infection. A shows pili or fibrillae protruding from the bacterial surface. These proteinaceous appendages bind to host cell surface molecules, usually carbohydrates, by adhesin proteins located at the distal tip of the pilus/fibrillar organelle. Antibodies targeting the adhesin protein block the bacterial/host interaction. B demonstrates a similar process of bacterial/epithelial cell interactions mediated by afimbrial adhesin proteins. In this case, antibodies directed against the bacterial surface proteins should also block attachment and colonization by impeding the ability of the bacteria to associate with mucosal tissues. C illustrates that some bacteria establish intimate associations with eukaryotic cells by intimin proteins, resulting in cytoskeletal rearrangements, host cell signaling, possible internalization of the bacteria, and in many cases systemic disease. Blocking the intimate association/adherence may also be another strategy to prevent bacterial infections. D shows a novel mechanism whereby bacteria secrete their own receptor protein, which is internalized by the target host cell, phosphorylated, and embedded in the eukaryotic cell as a new receptor for tight binding by the bacterium. Theoretically, blocking the secreted receptor (Hp90) before it is internalized by the host cell could provide another mechanism to block bacterial adherence and infection.

FimH adhesin produced by most *Enterobacteriaceae* (including uropathogenic *E. coli*), are highly conserved proteins (13). This lack of major variation is most likely due to the requirement that all pathogenic strains recognize invariant host receptors. Although minor changes in the adhesin protein have been observed (2% divergence) and correlate with decreased or increased affinity for binding to sugars (14), antibodies against a single FimH protein cross-react with >90% of *E. coli* strains expressing the FimH adhesin and block binding to bladder cells in vitro (15,16). Furthermore, antibodies against FimH from a single isolate protect against in vivo colonization by >90% of uropathogenic strains in a murine model for cystitis (unpub. obs.). This high degree of antigenic conservation is another reason why adhesins may serve as ideal vaccines.

Aside from mediating colonization of the host, bacterial attachment often results in the up-regulation or expression of many other virulence genes encoding various proteins that allow for invasion of the host (Figure 1C) (17-23). The proteins can mediate tighter associations with epithelial cells, trigger epithelial cell–actin filament rearrangements, and induce changes in host-cell signaling and function. In some cases, the bacteria may also secrete a protein that inserts into mammalian cells and serves as a receptor for its own intimate adherence with the host (Figure 1D) (24). Given that cross-talk between pathogenic bacteria and host cells after microbial attachment may trigger expression of virulence factors leading to local inflammation or invasive disease, vaccines that block bacterial attachment may have multiple advantages.

Many studies have demonstrated the utility of vaccines against bacterial surface components in blocking attachment in vitro as well as in vivo (25-28). However, as understanding of the mechanisms of attachment has evolved and characterization of specific adhesin molecules has been refined, new opportunities have emerged for the development of adhesin-based vaccines.

### Bacterial Adhesins: Gram-Negative Organisms

One of the best understood mechanisms of bacterial adherence is attachment mediated by cell surface structures called pili or fimbriae. Pili are long, flexible structures that extend outward from the bacterial surface of many species of bacteria and allow for contact between the bacteria and the host cell. Originally pili were thought to be homopolymeric structures composed of approximately 1,000 copies of a single structural subunit (fimbrin) packed in a helical array. However, for many pili, such as the highly characterized Type 1 and Pap pili expressed on *E. coli* and other *Enterobacteriaceae*, they are heteropolymeric structures with minor tip fibrillae proteins located at the distal end of the organelle (11). The specific interaction with receptor architectures on host cell surfaces is mediated by one of these tip proteins, called adhesins. For example, FimH adhesin mediates attachment to α-D-mannosides (29,30) by type 1 pili, while PapG mediates binding to α-D-gal(1-4)ß-D-Gal-containing receptors on host cells by Pap pili (31,32) (Table 1). The specificity of interaction may be involved in conferring tropism to the bladder and kidney tissues, respectively (33,38). Pilus-associated adhesins have been identified in a number of other bacteria as well (Table 1).

Not all adhesins are associated with pili. *Bordetella pertussis* expresses at least two

Table 1. Adhesins of gram-negative bacteria

| Adhesin | Strain | Ligand | Reference |
|---|---|---|---|
| Pili family[a] | | | Hultgren (33) |
| PapG | *Escherichia coli* | Gala(1-4)Gal in globoseries of glycolipids | |
| SfaS | *E. coli* | a-sialyl-2,3-b-galactose | |
| FimH | *E. coli* | Mannose-oligosaccharides | |
| HifE | *Haemophilus influenzae* | Sialylyganglioside-GM1 | |
| PrsG | *E. coli* | Gala(1-4)Gal in globoseries of glycolipids | |
| MrkD | *Klebsiella pneumoniae* | Type V collagen | |
| FHA | *Bordetella pertussis* | Sulfated sugars on cell-surface glycoconjugates | Brennan (34) |
| Pertactin | *B. pertussis* | Integrins | Brennan (34) |
| HMW1/HMW2 | *H. influenzae* | Human epithelial cells | St. Geme (35) |
| Hia | *H. influenzae* | Human conjunctival cells | Barenkamp (36) |
| $Le^b$-binding adhesin | *Helicobacter pylori* | Fucosylated $Le^b$ histo-blood group antigens | Ilver (37) |

[a]Representative examples from the large family of pilus-associated adhesins.
FHA, filamentous hemagglutinin; HMW, high molecular weight; Hia, *H. influenzae* adhesin putative adhesins on its surface: filamentous hemagglutinin (FHA) and pertactin (34). FHA is thought to mediate attachment to sulfated sugars on cell-surface glycoconjugates, although it may also have other properties. Pertactin is thought to mediate binding by the Arg-Gly-Asp triplet binding sequence characteristic of integrin-binding proteins, although the role of this binding activity in the pathogenesis of *Bordetella* infections is unclear. Both FHA and pertactin are components in the recently approved acellular pertussis vaccine.

In *Haemophilus influenzae,* two families of nonpilus adhesins have been identified: high-molecular weight adhesion proteins (HMW1 and HMW2) and immunogenic high molecular-weight surface-exposed proteins, the prototypic member of which has been designated Hia for *H. influenzae* adhesin (35). Both families are expressed by nontypable *H. influenzae,* which colonize the respiratory tract and cause such diseases as otitis media, pneumonia, and bronchitis. The HMW proteins, which share homology with the *B. pertussis* FHA protein, mediate specific attachment of *H. influenzae* to different types of human epithelial cells in vitro and have been implicated in directing respiratory tract tropism for these organisms. The Hia protein, in contrast, mediates tight association with human conjunctival cells and is present only in *H. influenzae* strains deficient in HMW1/HMW2 expression (36). Given the dichotomy among nontypable strains expressing either HMW or Hia-like adhesins and the serologic conservation at least among the HMW1 and HMW2 proteins, a vaccine based on a combination of such proteins may be protective against disease caused by most nontypable *H. influenzae*.

Another nonpilus adhesin has been identified and purified from *Helicobacter pylori*. The adhesin, called $Le^b$-binding adhesin, mediates bacterial adherence to fucosylated Lewis b ($Le^b$) histoblood group antigens, which are expressed along the mucosal surface of the gastric epithelium (37). The $Le^b$-binding adhesin may be involved in conferring tropism for stomach epithelium and allowing pathogenic bacteria to establish an ecologic niche within the gastrointestinal tract. In association with other virulence determinants expressed by *H. pylori* in the stomach, the colonization process ultimately results in ulcer formation.

A unique mechanism has been identified by which certain strains of enteropathogenic *E. coli* that cause severe diarrhea target cells for attachment: Enteropathogenic *E. coli* express and insert their own receptor (Hp90) into mammalian cell surfaces, thereby allowing the bacteria to attach and establish intimate contact with the epithelial cells (24) (Figure 1D). Although a bacterially encoded receptor for a cognate adhesin protein (intimin), Hp90 is expressed and secreted by enteropathogenic *E. coli* before colonization; therefore, it may also serve as a target for vaccine development.

### Bacterial Adhesins: Gram-Positive Organisms

Some of the most well-characterized colonization factors in gram-positive bacteria—the polypeptides of the antigen I/II family—bind to salivary glycoproteins in a lectinlike interaction (11) and promote adhesion to the tooth surface (Table 2). These proteins include the original AgI/II from *Streptococcus mutans*, also known as SpaP, P1, or PAc, and the *Streptococcus sobrinus* SpaA and PAg proteins (39). *Streptococcus gordonii* expresses two antigen I/II polypeptides, SspA and SspB, products of tandem chromosomal genes (39).

Surface proteins of the antigen I/II family contain alanine-rich repeats, which adopt an α-helical coiled-coil structure, proline rich repeats, and a carboxy-terminal region that includes the gram-positive cell wall anchor motif LPXTG (12,49). Binding activity to salivary glycoprotein has been attributed to both the highly conserved alanine rich repeats (50,51) and the proline-rich repeating sequences (52). In addition to salivary glycoprotein binding activity, SspA has been implicated in coaggregation of *S. gordonii* with Actinomyces (39). Such bacterial coaggregation may be involved in dental plaque formation.

Two other *S. gordonii* proteins, CshA and CshB, have also been implicated in coaggregation with Actinomyces. These adhesins may play a role in adherence of *S. gordonii* to immobilized fibronectin in vitro (52) and in colonization in vivo (40).

*Staphylococcus aureus* also expresses fibronectin-binding adhesins. Two genes encoding for fibronectin-binding proteins have been identified in *S. aureus*—*fnb*A and *fnb*B (41). Fibronectin binding activity is critical in

Table 2: Adhesins of gram-positive bacteria

| Adhesin | Strain | Ligand | Reference |
|---|---|---|---|
| Antigen I/II Family |  |  | Demuth (39) |
| Ag I/II, SpaP, P1, PAc | *Streptococcus mutans* | Salivary glycoprotein |  |
| SspA, SspB | *Streptococcus gordonii* | Salivary glycoprotein, Actinomyces |  |
| SpaA, PAg | *Streptococcus sobrinus* | Salivary glycoprotein |  |
| CshA , CshB | *S. gordonii* | Actinomyces, fibronectin | McNab (40) |
| FnbA, FnbB | *Staphylococcus aureus* | Fibronectin | Jonsson (41) |
| SfbI, Protein F | *Streptococcus pyogenes* | Fibronectin | Talay (42) Hanski (43) |
| LraI gamily |  |  | Burnett-Curley et al. (44) Jenkinson and Lamont (12) |
| FimA | *Streptococcus parasanguis* | Salivary glycoprotein fibrin |  |
| PsaA | *Streptococcus pneumoniae* | Unknown |  |
| ScaA | *S. gordonii* | Actinomyces |  |
| SsaB | *Streptococcus sanguis* | Salivary glycoprotein |  |
| EfaA | *Enterococcus faecalis* | Unknown |  |
| CbpA/SpsA/PbcA/ PspC | *S. pneumoniae* | Cytokine activated epithelial and endothelial cells, IgA | Rosenow (45) Hammerschmidt et al. (46) Briles et al. (47) Smith et al. (48) |

pathogenesis because it allows the bacteria to adhere to extracellular matrix components including fibronectin and collagen (53). This can result in cutaneous infections and in life-threatening bacteremia and endocarditis (54).

Binding to fibronectin is also essential for the attachment of *S. pyogenes* to respiratory endothelial cells. *S. pyogenes*, a group A streptococcus, has been implicated in various diseases from skin and throat infections to sepsis and shock (55). This binding activity is mediated by several fibronectin binding proteins. Six different genes encoding proteins with fibronectin binding activity have been identified (12). The most well characterized are two closely related proteins, SfbI (42) and Protein F (43). Both proteins are directly involved in the fibronectin-mediated adherence to epithelial cells.

Another group of extensively studied streptococcal adhesins is the LraI family of proteins. Included in this group are FimA from *S. parasanguis*, PsaA from *S. pneumoniae*, ScaA from *S. gordonii*, ScbA from *S. crista,* SsaB from *S. sanguis*, and *Efa*A from *Enterococcus faecalis* (44,56). These membrane-bound lipoproteins are part of a larger family of ATP-binding cassette metal permeases, involved in the acquisition of manganese (57).

The adhesins FimA and SsaB have high affinity for salivary glycoprotein on tooth surfaces and are involved in colonization of the oral cavity (58). The FimA adhesin for *S. parasanguis* has been localized to the tip of peritrichous surface fimbria on these bacteria (58). FimA is also a major virulence factor of *S. parasanguis* and binds fibrin (44). The ability to bind fibrin has been implicated in the pathogenesis of infective endocarditis.

The *S. pneumoniae* homologue of FimA, PsaA, also has a role in pathogenesis (59,60). While it commonly colonizes the nasopharynx of healthy persons, *S. pneumoniae* is a common pathogen in children and older adults and a leading cause of otitis media, bacterial pneumonia, sepsis, and meningitis. Like its homologues, PsaA may function as an adhesin, according to initial evidence (61). Insertion inactivation of *psaA* resulted in pneumococcal mutants that exhibited reduced adherence to alveolar epithelial cells in vitro (60). However, the PsaA protein may be a permease involved in the regulation of adherence rather than functioning as an adhesin per se (57,61).

Another pneumococcal protein thought to be an adhesin is CbpA (45) (also known as SpsA [46] and PspC [47]), one of a family of choline binding proteins (CBPs) (i.e., surface proteins noncovalently associated with the phosphocholine on the lipoteichoic acid). CbpA was initially isolated from a mixture of pneumococcal proteins that were able to bind to a choline affinity column. The CBP mix was purified from a strain with an inactivated *psp*A gene (45). The exogenous CBP mix inhibited adherence of pneumococci to type II pneumocytes and endothelial cells in vitro, suggesting that one or more of these proteins may act as adhesins. CbpA was the most abundant component in this mix and was shown to be on the surface of intact pneumococci in an indirect fluorescent-labeling assay. The CbpA protein also reacted strongly with a pool of human convalescent-phase serum. CbpA is thought to mediate adherence of *S. pneumoniae* to sialic acid and lacto-N-neotetraose ligands present on cytokine-activated epithelial and endothelial cells in vitro (45). *cbpA*-defective mutants did not colonize the nasopharynx of infant rats, further supporting its function as an adhesin and potential usefulness as an adhesin-based vaccine (45). In addition, others have shown that this choline binding protein (which they called SpsA) has IgA-binding properties, though the relevance of this function to pneumococcal pathogenesis is unclear (46). Yet another group has demonstrated complement protein C3-binding activity for this protein, which they termed PbcA (48). Whatever its role in pathogenesis, we have demonstrated that the gene for CbpA (SpsA/PbcA/PspC) is highly conserved among the most common pneumococcal isolates, further enhancing its use as a vaccine candidate (62).

### Adhesins as Vaccines: FimH as a Paradigm for Adhesin-Based Vaccines To Block Colonization

One of the key aspects of proving the potential efficacy of an adhesin-based vaccine in vivo is the development of an animal model of disease that relies on bacterial colonization of the mucosal epithelium mediated by the specific adhesin of interest. Although seemingly straightforward, testing for protection in small animal models of disease is difficult for various reasons: large doses of in vitro grown bacteria are required to establish mucosal colonization in animals, which does not necessarily mimic the course of infection in humans; specific glycoprotein receptors for some adhesins are lacking in animal mucosal tissues that correspond to the site of colonization in humans; and some bacterial adhesins that are usually expressed as part of a larger structure on the bacterial cell surface (e.g., tip adhesins associated with whole pili) are difficult to purify. Despite these difficulties, adhesin-based vaccines have demonstrated efficacy in protecting against infection, thus proving the usefulness of such molecules as subunit vaccines. Research using the FimH adhesin from *E. coli* provides one such example.

Type 1 pili have long been implicated in bacterial urinary tract infections in humans (63,64). In a murine cystitis model, colonization of the bladder by *E. coli* was shown to depend on growth conditions that favored expression of type 1 pili and in particular required FimH (15,65). Thus, the murine model was a valid small-animal model to prove whether adhesin-based vaccines might block colonization.

Although purifying large amounts of pilus-associated adhesin is difficult (because most adhesins are proteolytically degraded when expressed as independent moieties), Hultgren et al. demonstrated that the FimH adhesin could be stabilized in an active conformation by the periplasmic chaperone FimC, making it possible to purify full-length FimH protein. Vaccination with the FimCH complex elicited long-lasting immune responses to FimH. Sera from mice vaccinated with the FimH vaccine inhibited uropathogenic strains of *E. coli* from binding to human bladder cells in vitro. Vaccination with the FimH adhesin-vaccine reduced in vivo colonization of the bladder mucosa by >99% in the murine cystitis model (15). Furthermore, the FimH vaccine protected against colonization and disease by uropathogenic strains of *E. coli* capable of expressing multiple adhesins. IgG specific for FimH was detected in the urine of protected mice, consistent with our original hypothesis that antibodies directed against an adhesin protein might protect along the mucosal surface. Subsequent studies in a primate model of cystitis have corroborated these findings (Langermann et al., unpub. data). Furthermore, in primate studies we demonstrated a direct correlation between the presence of inhibitory antibodies in secretions and protection against colonization and infection.

While IgG antibodies elicited against adhesins are protective, induction of immune responses along the mucosa can be augmented by a variety of antigen delivery systems that specifically target mucosa-associated lymphoid tissue and activate the mucosal immune system (4-7,66). These delivery systems include whole-inactivated or live-attenuated bacterial and viral vectors, biodegradable microspheres, liposomes, transgenic plants, and antigens conjugated to or coadministered with the cholera toxin B subunit or attenuated forms of heat labile toxin from *E. coli*. Many of these systems hold promise for future vaccine strategies, but only a few have been tested in humans for safety and adjuvanticity. As these mucosal adjuvants progress further toward approval for use in humans, testing should be done with adhesin antigens to determine if induction of local immune responses enhances the protective efficacy of adhesin-based vaccines as compared with conventional parenteral vaccination. Such studies are under way with the FimH vaccine.

Given the preclinical data with the FimH vaccine, similar efforts should be directed at developing adhesin-based vaccines for a wide range of pathogens. In this regard, additional efforts should also be focused on developing mucosal models of infection. The availability of such models should allow for appropriate screening of adhesin-based vaccines to prevent infections by streptococci, staphylococci, and other pathogens for which vaccine coverage is absent or inadequate.

### Acknowledgments

We thank Luis Branco for his illustration (Figure 1) and Scott Koenig and Jennifer Bostic for reviewing the manuscript.

Dr. Wizemann served as *Streptococcus pneumoniae* Vaccine Project Team Leader at MedImmune from 1996-98.

### References

1. Hilleman MR. Paper presented at the International Symposium on Recombinant Vectors in Vaccine Development; 1993 May 23; Albany, New York. [Nucleic Acids Technologies Foundation, sponsored by IABS, FDA, USDA/APHIS, and NIAID/NIH].
2. Alexander J, Fikes J, Hoffman S, Franke E, Sacci J, Appella E, Chisari, FV, et al. The optimization of helper T lymphocyte (HTL) function in vaccine development. Immunol Res 1998;18:79-92.
3. St Geme JW III. Bacterial adhesins: determinants of microbial colonization and pathogenicity. Adv Pediatr 1997;44:43-72.
4. McGhee J, Mestecky J, Dertzbaugh MT, Eldridge JH, Hirasawa M, Kiyono H. The mucosal immune system: from fundamental concepts to vaccine development. Vaccine 1992;10:75-88.
5. Langermann S. New approaches to mucosal immunization. Semin Gastrointest Dis 1996;7:12-8.
6. O'Hagan DT. Recent advances in vaccine adjuvants for systemic and mucosal administration. J Pharm Pharmacol 1998;50:1-10.
7. Mestecky J, Michalek SM, Moldoveanu Z, Russell MW. Routes of immunization and antigen delivery systems for optimal mucosal immune responses in humans. Behring Inst Mitt 1997;98:33-43.
8. Beachey EH. Bacterial adherence: adhesin-receptor interactions mediating the attachment of bacteria to mucosal surface. J Infect Dis 1981;143:325-45.
9. Beachey EH, Giampapa CS, Abraham SN. Bacterial adherence: adhesin receptor-mediated attachment of pathogenic bacteria to mucosal surfaces. American Review of Respiratory Diseases 1988;138:S45-8.
10. Ofek I, Sharon N. Adhesins as lectins: specificity and role in infection. Curr Top Microbiol Immunol 1990;151:91-113.
11. Hultgren SJ, Abraham S, Capron M, Falk P, St. Geme JW, Normark S. Pilus and non-pilus bacterial adhesins: assembly and function in cell recognition. Cell 1993;73:887-901.
12. Jenkinson HF, Lamont RJ. Streptococcal adhesion and colonization. Crit Rev Oral Biol Med 1997;8:175-200.
13. Abraham SN, Sun D, Dale JB, Beachey EH. Conservation of the D-mannose-adhesion protein among type 1 fimbriated members of the family *Enterobacteriacaea*. Nature 1988;682-4.
14. Sokurenko EV, Courtney HS, Ohman DE, Klemm P, Hasty DL. FimH family of type 1 fimbrial adhesins: functional heterogeneity due to minor sequence variations among *fim*H genes. J Bacteriol 1994;176:748-55.
15. Langermann S, Palaszynski S, Barnhart M, Auguste G, Pinkner JS, Burlein J, et al. Prevention of mucosal *Escherichia coli* infection by FimH-based systemic vaccination. Science 1997;276:607-11.
16. Palaszynski S, Pinkner J, Leath S, Barren P, Auguste CG, Burlein J, et al. Systemic immunization with conserved pilus-associated adhesins protects against mucosal infections. Dev Biol Stand 1998;92:117-22.
17. Finlay B, Falkow S. Common themes in microbial pathogenicity. Microbiological Review 1989;53:210-30.
18. Hoepelman AI, Tuomanen EI. Consequences of microbial attachment: directing host cell functions with adhesins. Infect Immun 1992;60:1729-33.
19. Svanborg C, Hedlund M, Connell H, Agace W, Duane, RD, Nilsson A, et al. Bacterial adherence and mucosal cytokine responses. Receptors and transmembrane signaling. Ann N Y Acad Sci 1996;797:177-90.
20. Falkow S. Invasion and intracellular sorting of bacteria: searching for bacterial genes expressed during host/pathogen interactions. J Clin Invest 1997;100:239-43.
21. Mecsas JJ, Strauss EJ. Molecular mechanisms of bacterial virulence: type III secretion and pathogenicity islands. Emerg Infect Dis 1996;2:270-88.
22. Zhang JP, Normark S. Induction and gene expression in *Escherichia coli* after pilus-mediated adherence. Science 1996;273:1234-6.

23. Donnenberg MS, Kaper JB, Finlay BB. Interactions between enteropathogenic *Escherichia coli* and host epithelial cells. Trends Microbiol 1997;5:109-14.
24. Kenny B, DeVinney R, Stein M, Reinscheid DJ, Frey EA, Finlay BB. Enteropathogenic *E. coli* (EPEC) transfers its receptor for intimate adherence into mammalian cells. Cell 1997;91:511-20.
25. Svanborg-Eden C, Marild S, Korhonen TK. Adhesion inhibition by antibodies. Scand J Infect Dis 1982;33:72-8.
26. O'Hanley P, Lark D, Falkow S, Schoolnik G. Molecular basis of *Escherichia coli* colonization of the upper urinary tract in Balb/c mice. Gal-Gal pili immunization prevents *Escherichia coli* pyelonephritis in the Balb/c mouse model of human pyelonephritis. J Clin Invest 1985;75:347-60.
27. Pecha B, Low D, O'Hanley P. Gal-Gal pili vaccines prevent pyelonephritis by piliated *Escherichia coli* in a murine model. Single component Gal-Gal pili vaccines prevent pyelonephritis by homologous and heterologous piliated *E. coli* strains. J Clin Invest 1989;83:2102-8.
28. Moon HW, Bunn TO. Vaccines for preventing enterotoxigenic *Escherichia coli* infections in farm animals. Vaccine 1993;11:213-20.
29. Maurer L, Orndorff P. Identification and characterization of genes determining receptor binding and pilus length of *Escherichia coli* type 1 pili. J Bacteriol 1987;169:640-5.
30. Hanson MS, Brinton CC Jr. Identification and characterization of the *Escherichia coli* type 1 pilus adhesin protein. Nature 1988;322:265-8.
31. Bock K, Breimer ME, Brignole A, Hansson GC, Karlsson KA, Larson,G, et al. Specificity of binding of a strain of uropathogenic *Escherichia coli* to Gal 1-4Gal-containing glycosphingolipids. J Biol Chem 1985;260:8545-51.
32. Stromberg N, Marklund BI, Lund B, Ilver D, Hamers A, Gaastra W, et al. Host-specificity of uropathogenic *Escherichia coli* depends on differences in binding specificity to Gal 1-4Gal-containing isoreceptors. EMBO J 1990;9:2001-10.
33. Hultgren SJ, Jones CH. Utility of the immunoglobulin-like fold of chaperones in shaping organelles of attachment in pathogenic bacteria. American Society for Microbiology News 1195;61:457-64.
34. Brennan MJ, Shahin RD. Pertussis antigens that abrogate bacterial adherence and elicit immunity. Am J Respir Crit Care Med 1996;154:S145-9.
35. St. Geme JW III. Progress towards a vaccine for nontypable *Haemophilus influenzae*. The Finnish Medical Society DUODECIM. Ann Med 1996;28:31-7.
36. Barenkamp SJ, St Geme JW III. Identification of a second family of high-molecular-weight adhesion proteins expressed by non-typable *Haemophilus influenzae*. Mol Microbiol 1996;19:1215-23.
37. Ilver D, Arnqvist A, Ogren J, Frick IM, Kersulyte D, Incecik ET, et al. *Helicobacter pylori* adhesin binding fucosylated histo-blood group antigens revealed by retagging. Science 1998;279:373-7.
38. Roberts JA. Tropism in bacterial infections: urinary tract infections. J Urol 1996;156:1552-9.
39. Demuth DR, Duan Y, Brooks W, Holmes AR, McNab R, Jenkinson HF. Tandem genes encode cell-surface polypeptides SspA and SspB which mediate adhesion of the oral bacterium *Streptococcus gordonii* to human and bacterial receptors. Mol Microbiol 1996;20:403-13.
40. McNab R, Jenkinson HF, Loach DM, Tannock GW. Cell-surface-associated polypeptides CshA and CshB of high molecular mass are colonization determinants in the oral bacterium *Streptococcus gordonii*. Mol Microbiol 1994;14:743-5.
41. Jonsson K, Signas C, Muller HP, Lindberg M. Two different genes encode fibronectin binding proteins in *Staphylococcus aureus*. The complete nucleotide sequence and characterization of the second gene. Eur J Biochem 1991;202:1041-8.
42. Talay SR, Valentin-Weigand P, Timmis KN, Chhatwal GS. Domain structure and conserved epitopes of Sfb protein, the fibronectin-binding protein adhesin of *Streptococcus pyogenes*. Mol Microbiol 1994;13:531-9.
43. Hanski E, Caparon M. Protein F, a fibronectin-binding protein, is an adhesin of the group A streptococcus *Streptococcus pyogenes*. Proc Natl Acad Sci U S A 1992;89:6172-6.
44. Burnette-Curley D, Wells V, Viscount H, Munro CL, Fenno JC, Fives-Taylor P, et al. FimA, a major virulence factor associated with *Streptococcus parasanguis* endocarditis. Infect Immun 1995;63:4669-74.
45. Rosenow C, Ryan P, Weiser JN, Johnson S, Fontan P, Ortqvist A, et al. Contribution of novel choline-binding proteins to adherence, colonization and immunogenicity of *Streptococcus pneumoniae*. Mol Microbiol 1997;25:819-29.
46. Hammerschmidt S, Talay SR, Brandtzaeg P, Chhatwal GS. SpsA, a novel pneumococcal surface protein with specific binding to secretory immunoglobulin A and secretory component. Mol Microbiol 1997;25:1113-24.
47. Briles DE, Hollingshead SK, Swiatlo E, Brooks-Walter A, Szalai A, Virolainen A, et al. PspA and PspC: their potential for use as pneumococcal vaccines. Microb Drug Resist 1997;3:401-8.
48. Smith BL, Cheng Q, Hostetter MK. Characterization of a pneumococcal surface protein that binds complement protein C3 and its role in adhesion [poster D-122]. The American Society for Microbiology (1998) 98th General Meeting; Atlanta, Georgia.
49. Hajishengallis G, Russell MW, Michalek SM. Comparison of an adherence domain and a structural region of *Streptococcus mutans* antigen I/II in protective immunity against dental caries in rats after intranasal immunization. Infect Immun 1998;66:1740-3.
50. Crowley PJ, Brady LJ, Piacentini DA, Bleiweis AS. Identification of a salivary agglutinin-binding domain within cell surface adhesin P1 of *Streptococcus mutans*. Infect Immun 1993;61:1547-52.
51. Todryk SM, Kelly CG, Lehner T. Effect of route of immunisation and adjuvant on T and B cell epitope recognition within a streptococcal antigen. Vaccine 1998;16:174-80.
52. McNab R, Holmes AR, Clark JM, Tannock GW, Jenkinson HF. Cell surface polypeptide CshA mediates binding of *Streptococcus gordonii* to other oral bacteria and to immobilized fibronectin. Infect Immun 1996;64:4202-10.

53. Schennings T, Heimdahl A, Coster K, Flock J-I. Immunization with fibronectin binding protein from *Staphylococcus aureus* protects against experimental endocarditis in rats. Microb Pathog 1993;15:227-36.
54. Lee JC. The prospects for developing a vaccine against *Staphylococcus aureus*. Trends Microbiol 1996;4:162-6.
55. Molinari G, Talay SR, Valentin-Weigand P, Rohde M, Chhatwal GS. The fibronectin-binding protein of *Streptococcus pyogenes*, SfbI, is involved in the internalization of group A streptococci by epithelial cells. Infect Immun 1997;65:1357-63.
56. Correia FF, DiRienzo JM, McKay TL, Rosan B. *scb*A from *Streptococcus crista* CC5A: an atypical member of the *lra*I gene family. Infect Immun 1996;64:2114-21.
57. Dintilhac A, Alloing G, Granadel C, Claverys JP. Competence and virulence of *Streptococcus pneumoniae*: Adc and PsaA mutants exhibit a requirement for Zn and Mn resulting from inactivation of putative ABC metal permeases. Mol Microbiol 1997;25:727-39.
58. Fenno JC, Shaikh A, Spatafora G, Fives-Taylor P. The *fim*A locus of *Streptococcus parasanguis* encodes an ATP-binding membrane transport system. Mol Microbiol 1995;15:849-63.
59. Talkington DF, Brown BG, Tharpe JA, Koenig A, Russell H. Protection of mice against fatal pneumococcal challenge by immunization with pneumococcal surface adhesin A (PsaA). Microb Pathog 1996;21:17-22.
60. Berry AM, Paton JC. Sequence heterogenity of PsaA, a 37-kilodalton putative adhesin essential for virulence of *Streptococcus pneumoniae*. Infect Immun 1996;64:5255-62.
61. Novak R, Brown JS, Charpentier E, Tuamonen E. Penicillin tolerance genes of *Streptococcus pneumoniae*: the ABC-type manganese permease complex Psa. Mol Microbiol 1998;29:1285-96.
62. Dormizter M, Wizemann TM, Adamou JE, Walsh W, Gayle T, Langermann S, et al. Sequence and structural analysis of CbpA, a novel choline-binding protein of *Streptococcus pneumoniae* [poster B-3]. The American Society for Microbiology (1998) 98th General Meeting; Atlanta, Georgia.
63. Schaeffer AJ, Amundsen SK, Schnidt LN. Adherence of *Escherichia coli* to human urinary tract epithelial cells. Infect Immun 1979;24:753-9.
64. Ofek I, Mosek A, Sharon N. Mannose-specific adherence of *Escherichia coli* freshly excreted in the urine of patients with urinary tract infections, and of isolates subcultured from the infected urine. Infect Immun 1981;34:708-11.
65. Connell H, Agace W, Klemm P, Schembri M, Marild S, Svanborg C. Type 1 fimbrial expression enhances *Escherichia coli* virulence for the urinary tract. Proc Natl Acad Sci U S A 1996;93:9827-32.
66. Frey A, Neutra MR. Targeting of mucosal vaccines to Peyer's patch M cells. Behring Inst Mitt 1997;98:376-89.
